# A Multicomponent Primary-Care Intervention for Preventing Falls in Older Adults Living in the Community: The PREMIO Study

**DOI:** 10.3390/jcm12227134

**Published:** 2023-11-16

**Authors:** Walter Marrocco, Antonella Galli, Silvestro Scotti, Nicola Calabrese, Paolo Misericordia, Alessandro Dalle Vedove, Gianmarco Marrocco, Antonio Pio D’Ingianna, Andrea Pizzini, Massimo Fini, Carlo Tomino, Stefano Bonassi, on behalf of the F.I.M.M.G Research PREMIO Group

**Affiliations:** 1 Federazione Italiana Medici di Medicina Generale—F.I.M.M.G. (Italian Federation of General Practitioners), 00125 Rome, Italy; dott.antonellagalli@gmail.com (A.G.); silvestroscotti@gmail.com (S.S.); nicorese@gmail.com (N.C.); p.misericordia@gmail.com (P.M.); gianmarco.marrocco90@gmail.com (G.M.); adi.altomonte@tiscalinet.it (A.P.D.); andrea.pizzini09@gmail.com (A.P.); 2 Società Italiana di Medicina di Prevenzione e degli Stili di Vita—S.I.M.P.e.S.V. (Italian Society of Preventive Medicine and Lifestyle), 00125 Rome, Italy; 3 NETMEDICA ITALIA, 00125 Rome, Italy; alessandro.dallevedove@netmedicaitalia.it; 4 IRCCS San Raffaele, 00166 Rome, Italy; massimo.fini@sanraffaele.it (M.F.); carlo.tomino@sanraffaele.it (C.T.); stefano.bonassi@sanraffaele.it (S.B.); 5 Department of Human Sciences and Quality of Life Promotion, San Raffaele University, 00166 Rome, Italy

**Keywords:** falls, prevention, fractures, elderly people

## Abstract

Background: Falls are a common cause of morbidity and functional impairment in the elderly and represent a significant health problem. General practitioners (GPs) are the first point of contact for health issues and may provide preventive services. The randomized clinical trial PREMIO was conducted by GPs to evaluate the effects of a multicomponent intervention for the prevention of falls in older adults aged ≥ 65 years at high risk of falling. Methods: 117 GPs enrolled 1757 patients (1116 F, 641 M) and randomized them into 2 groups (intervention and control). The intervention group received medical and behavioral counseling, home risk-factor assessment, a physical-activity program and nutritional counseling. The control group received only the nutritional counseling. Both groups were followed for one year. The primary outcome was the rate of falls at home over 12 months. Results: 1225 patients completed the study. Subjects receiving the intervention had, on average, fewer falls at home (percentage change −31.2%, *p* < 0.02) and fewer total falls (−26.0%, *p* < 0.02), although the reduction in the number of fallers was small (−3.9%, *p* = 0.05). Among the secondary endpoints, rates of general hospital or emergency-department admission and GP visits showed slight improvements (not statistically significant), while the risk of fractures was unexpectedly increased in the intervention group compared to the controls (odds ratio 2.39, *p* = 0.023). Conclusions: Future studies and public-health interventions to prevent domestic falls among community-dwelling older people at high risk of falling could benefit from a multicomponent approach including medication review, physical exercise and home risk assessment and should include assessment of risk factors for fractures.

## 1. Introduction

Falls are a common cause of morbidity and functional impairment in older people and represent a significant health problem that requires preventive action.

It is estimated that approximately one in three people aged 65 years and over and half of those aged 80 and over experience at least one fall per year [1]. As the frequency of this event increases with increasing age, the magnitude of the problem will increase as the population ages [2].

Injuries resulting from falls are often not serious, but 10% to 20% of fallers aged 65 years and older sustain bone fractures [3]. Globally, among people aged ≥ 70 years, falls result in more than 6 million years lived with disability, and 1.6% (95% CI 1.32–1.79) of falls result in death [4]. Falls in the elderly represent a major concern because of their impact on public health and can result in a significant economic burden because of the substantial associated medical expenditures [5,6].

Among older people, experiencing falls has been identified as a risk factor for nursing-home admission [7], and repeated falls are a common reason for admission to the hospital [8]. Loss of confidence following a fall can have psychological consequences for older people, who may be afraid of falling again. This change can lead to reduction in physical activity, depression and social isolation, accelerating functional decline [9].

Recognized fall risk factors related to the health status of the elderly include poor health [10], conditions due to cardiovascular diseases [11,12], depression [13], dementia [14], arthritis [15], epilepsy [16] and visual impairment [17]. Polypharmacy and the use of certain medications, such as benzodiazepine, psychotropics and some classes of cardiovascular drugs, can also increase the risk of falling [12,18,19,20].

In addition to individual health-related risk factors, environmental hazards also play a role in many falls. These hazards include slippery floors or stairs; inadequate, irregular or excessive lighting; and lack of handholds [21]. 

The ongoing Silver Steps (Passi d’Argento) survey, promoted by the Italian National Institute of Health (ISS), provides an overview of the situation in Italy [22]. In 2012–2013, 11.2% (CI 95% 10.64–11.69%) of respondents aged 65 years and over (*n* = 24.000) reported having fallen in the 30 days prior to the interview, with 14% (CI 95% 12.4–15.8%) of cases requiring at least one day of hospitalization [23]. Most falls occurred in the home (60.5%, CI 95% 58.2–62.7%), with falls representing the leading cause of home accidents. Falls occurred less frequently in the street (19.5%, CI 95% 17.8–21.2%) or elsewhere. The survey also found that only one in six respondents said they had received advice from their doctor or other healthcare professional about how to prevent falls in the 12 months prior to the survey, suggesting a need for greater awareness and preventive interventions. Multivariate analysis identified several factors that showed statistically significant associations with increased risk of falling, including age, female gender, disability, polypharmacy, visual impairment, economic difficulties and living in a house with structural problems.

Falls are caused by multiple factors, so any prevention strategy should be based on interventions with multiple components [24]. The recent global guidelines for fall prevention in older adults recommend multicomponent interventions, defined as “fixed combinations of two or more intervention components that are not individually tailored following a multifactorial falls risk assessment. Multicomponent interventions vary widely: for illustration, an example could be a medication review, home modifications and generic exercise advice” [25]. Recommendations for healthy nutrition should be included, as both nutritional status and BMI are associated with the risk of falls and recurrent falls [26]. 

General practitioners (GPs) are the first point of contact for health issues. They are in the best position to assess the presence of risk factors for falls, to provide fall-prevention interventions to community-dwelling older adults, and to evaluate the impact of prevention strategies. In Italy, GPs are present in the community, their clinics are easily accessible to patients, and they conduct clinical practice both in their clinic and in the patient’s home. The GP thus has easy access to the home environment to assess safety conditions and daily habits. In addition, GPs can provide continuity of care over time and have the capacity to follow up on long-term multicomponent interventions.

The PREMIO study (Primary Prevention Study of Domiciliary Falls in Elderly Patients at Risk, in Italian: Studio di PREvenzione PriMaria delle Cadute DomIciliari in Pazienti Anziani a rischiO) was launched in 2015 by one of the largest Italian federations of GPs (in Italian: Federazione Italiana Medici di Medicina Generale-FIMMG). The aim of the study was to implement a multicomponent preventive intervention program coordinated by the GPs and to evaluate its impact on the rate of reported falls and their serious complications such as fractures and fall-related healthcare utilization. The effect of this intervention was compared with the effect of simple dietary recommendations.

## 2. Materials and Methods

### 2.1. Participants

The first 20 consecutive patients who met the inclusion criteria and agreed to participate in the study were recruited by their respective GPs during clinic or home visits. Inclusion criteria required the presence of at least five of the following fall risk factors: history of previous falls, fear of falling, polypharmacy (≥5 medications), treatment with medications that increase the risk of falling (Table S1), impaired mobility, altered vision, social isolation, major cerebro- or cardiovascular disease, difficulty extending the knees, mental confusion, creatinine clearance < 65 mL/min and arthritis and/or arthrosis. The five-factor threshold was arbitrarily defined as a reliable compromise for sample enrichment that was helpful in identifying a population who were at high risk for falls but also fit enough to actively participate in a low-intensity program of physical activity.

Other inclusion criteria were age ≥ 65 years, living at home regularly and signing the informed consent. Individuals with a life expectancy of <1 year; those with Parkinson’s disease, epilepsy or depression (under antidepressant treatment); bedridden patients and, in general, all subjects with serious psychophysical conditions that prevented their participation in the study were excluded from the selection procedures.

### 2.2. Study Design

The PREMIO study was a two-arm, pragmatic, randomized controlled trial. All GPs identified through their membership in the national medical organization FIMMG were invited to participate in the study through presentations, e-mails, letters and face-to-face interviews. A total of 117 GPs agreed to participate, and each doctor committed to enrolling 20 patients who were considered at risk of falling, according to the then-current Italian guidelines for the prevention of falls at home among the elderly [27]. 

Patients were randomized using a sealed-envelope system. Participating GPs received randomly generated treatment allocations in sealed opaque envelopes. The envelope was opened once a patient who met the inclusion criteria agreed to participate in the program.

All patients randomized to the intervention group received a booklet with physical-activity recommendations (Table S2).

They also received a Fall Diary (Table S3) and a Physical Activity Diary (Table S4), both to be completed daily and sent to the GP every three months. To complete the intervention, the patients received a set of dietary recommendations (Table S5).

The patients in the control arm received the same set of dietary recommendations (as required by the ethics committee) and the Fall Diary.

For each enrolled patient, the GP completed a patient data sheet with demographic and medical data based on the patient’s clinical records. These data and the information from the fall and physical-activity diaries were centralized through a web platform for the creation and management of the PREMIO database at NETMEDICA (www.netmedicaitalia.it, accessed on 20 October 2023).

Within 2 weeks of enrollment, the GP was to visit each patient’s home, identify home risks and provide recommendations to address them, and complete the environmental risk survey module (Table S6). All GPs contacted the patients monthly to monitor compliance and reinforce motivation to participate in the study.

The intervention lasted 12 months. At the end of the study, all participating patients were asked to complete a customer-satisfaction questionnaire based on a 7-item Likert scale (Table S7) to assess the acceptability of the program.

### 2.3. Intervention

The intervention plan was multicomponent and included the following:

- medical review of treatments, with the aim of limiting medications that increase the risk of falling (Table S1)
- recommendation of 1–2 daily training sessions with gentle physical exercise (5 min of stationary exercise plus 5 min of slow walking and 5 min of fast walking, gradually increasing up to 30 min, followed by 5 min of slow walking. See Table S2)
- inspection of patients’ homes, followed by recommendations of home modifications to reduce structural hazards (e.g., installing a handrail on stairs or equipping the shower stall or bathtub with non-slip mats. See Table S6).

Minor interventions added to the program at the discretion of the GP included, for example, management of orthostatic hypotension, podiatry, diet and practical recommendations (e.g., safe shoe models).

### 2.4. Outcomes

The primary outcome was the rate of falls over 12 months. To investigate different characteristics of fallers, fall rates were expressed as both the mean number of falls and the proportion of individuals reporting at least one fall.

Secondary outcomes included the direct consequences of falls, i.e., fractures, admissions to the hospital or emergency department and visits to their GP’s clinic.

### 2.5. Ethical Compliance

The study protocol was approved by the Ethics Committee of the coordinating center at the IRCCS San Raffaele Roma (PR n. 06/16; 10 March 2016) and by the ethics committees of the local health districts where the GPs were located (over 40 ethics committees were contacted).

All participating subjects signed an informed-consent form after receiving detailed information about the aims of the study and the protocol.

### 2.6. Statistical Methods

The study was designed to show a significant (5%) reduction in falls in the intervention group, that is, a change from an expected frequency of 27% per year to 22%, considering a type-I error of 0.05 and a statistical power of 80%. The estimated number of patients to recruit was 953 per group. The G*power statistical software was used for power analysis.

The primary statistical analysis was based on 1757 individuals who started the study. The protocol, which required participants to keep records of physical activity and falls over the whole duration of the study, was completed by 1225 individuals (69.7%).

A flow chart describing patient dropout and withdrawal is provided in Supplementary Figure S1. The mean age, sex ratio, and the mean of fall risk factors were compared between the subjects who left the study and those who completed the program. No significant difference was found for any parameters evaluated.

The presence of differences in clinical and demographic characteristics between treatment arms was tested with Student’s *t*-test for continuous variables and with the χ^2^ test for categorical variables.

The effect of the intervention on the number of falls was evaluated with a log-normal multiple regression model and with logistic regression models for binary variables. Risks for all endpoints were evaluated as mean ratio (MR) and odds ratio (OR), depending on the model fitted, and reported with their 95% confidence intervals (95% CI).

All statistical analyses were performed using SPSS version 23 (IBM Corp. Released 2015. IBM SPSS Statistics for Windows, version 23.0. Armonk, NY, USA: IBM Corp), and Stata version 15.1 (StataCorp. 2017. College Station, TX, USA: StataCorp LLC).

## 3. Results

The PREMIO study involved 117 GPs who enrolled 1757 patients at high risk of falling. Half of the patients were randomized to intervention and half to the control group, as shown in Table 1.

Patients were predominantly female (63.5%), and the mean age of the entire study group was 77.5 years. Three out of four patients were overweight or obese (45.2% and 29.4%, respectively), while only 0.9% were underweight.

Current smokers constituted 17.1% of the total study group, although information on smoking habits was largely underreported in the medical records (47.7% missing). Smoking habits was the only variable with such a high proportion of missing values. There were no statistically significant differences between the two study groups in these selected characteristics, confirming the effectiveness of randomization. The difference in the number of patients randomized to the intervention and control groups resulted from an unequal distribution of patients recruited by each GP.

The distribution of fall risk factors among the patients is shown in Table 2.

One of the inclusion criteria was having at least 5 risk factors from the predefined list. The mean number of risk factors was 6; the most common number was 5 (66%), followed by 6 (19%). The use of medications that increase the risk of falling was the most common risk factor (93.5%), followed by cerebro- or cardiovascular disease (87.6%), polypharmacy (85.5%) and the presence of arthritis or arthrosis (85.5%), this last being the only factor that showed a small but statistically significant difference between the intervention and control groups.

To test whether selection bias was introduced by differential attrition of subjects from the multicomponent intervention group versus the control group, the proportions of subjects who completed the protocol and recorded physical activity and falls for the full 12 months of the study were compared in the two groups and were found to substantially overlap (68.6% and 66%, respectively). The main reason for patient withdrawal was the progressive loss of interest in the study. Other common reasons included the GP’s decision to leave the study, health reasons including death and practical reasons such as change of residence or change of GP.

Table 3 shows the distribution of patients according to selected characteristics of the 117 GPs who participated in the study.

Three out of four patients had male GPs (76.4%). The mean age of the GPs was 59.4 years. Almost half were from southern Italy (52.5%), and the rest were from central-northern Italy. On average, each doctor enrolled 15 patients. No statistically significant difference was found between the two study groups for any of these characteristics.

In the intervention group, most patients had their GPs make the two planned home visits (81.4%), while the rest made only one; 80% had regular telephone contact with patients.

Table 4 shows the results of the univariate analysis of primary endpoints.

Subjects who received the multicomponent intervention had a lower mean number of falls (−26.0%, *p* < 0.02). The reduction was more dramatic in the subgroup of domestic falls, the target of the intervention (−31.2%, *p* < 0.02) but did not reach statistical significance in the subgroup of falls outside. In the subgroup of domestic falls, the number of fallers was also slightly lower in the intervention group than in the control group (*p* = 0.05).

Multivariate models were used to account for residual confounding and to test for the presence of interactions. The log-normal multivariate model fitted to the mean number of falls showed a significantly lower mean number of domestic falls in the multicomponent-intervention group compared with the controls (mean ratio 0.70, 95% CI: 0.54–0.90; *p* = 0.032) after adjustment for the potential confounders of age, sex, BMI and smoking habits.

Binary logistic regression analysis of the risk of falling at least once per year showed an odds ratio (OR) of 0.76 (95% CI: 0.57–1.02; *p* = 0.068), which was estimated from a model including age and sex.

**Figure 1:**
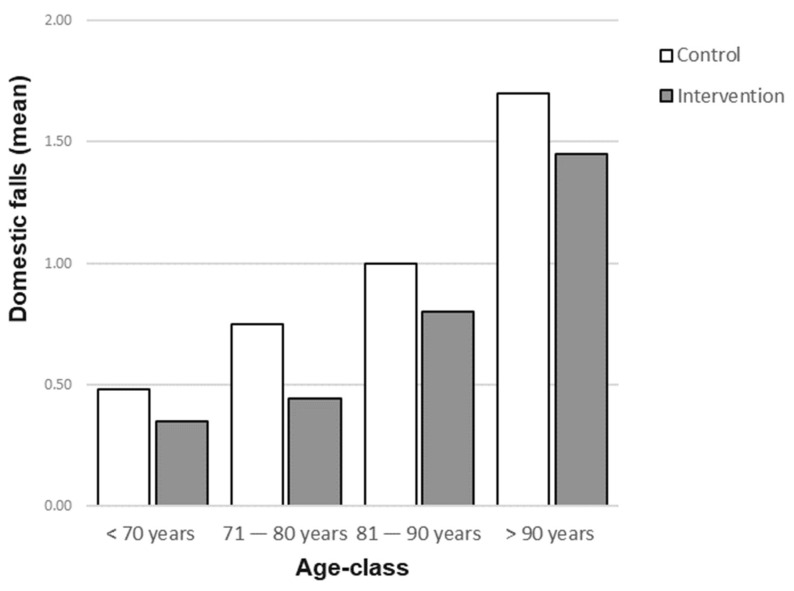
Mean number of domestic falls by PREMIO study group and by age-class.

No interaction was found between the effect of the multicomponent intervention and other covariates, although some covariates were closely associated with the risk of falling. Figure 1 shows that the mean number of domestic falls increased with age in both the intervention and control groups but was consistently lower in the intervention group than in the control group across all age classes.

The study included several secondary endpoints, as summarized in Table 5.

The overall frequency of fractures due to falls was 2.7%, and, unexpectedly, subjects in the intervention group showed a higher risk compared to controls (OR = 2.39, *p* = 0.023).

No statistically significant differences were observed for the other endpoints, although subjects in the intervention arm were less likely to be admitted to hospitals or to emergency departments and were less likely to visit the GPs’ clinics (16.5% of subjects overall, for a total of 764 visits).

Patients in the intervention arms were given a questionnaire to assess their level of satisfaction with the fall-prevention program. The mean score was 6.4 (SD 1.3), which corresponds to complete satisfaction on a 0–7 Likert scale.

## 4. Discussion

The PREMIO study involved engaging many GPs across the country to raise awareness of the problem of falls in older adults and propose an active role for the primary care physician. The multicomponent intervention proposed here was supported by extensive evidence, and our results confirm the validity and effectiveness of this approach.

Patients randomized to the multicomponent intervention experienced a statistically significant reduction in the mean number of falls recorded during 12 months of follow-up (−26.0% overall). The reduction was particularly dramatic for domestic falls, which were reduced by 31.2% (mean number of falls per year: 0.80 in the control group vs. 0.55 in the intervention group). The overall number of fallers was reduced by 3.9% (*p* = 0.05).

Limited improvement was also seen in other secondary outcomes of great importance for public health, such as admission to a general hospital (2.3% in the intervention group vs. 2.6% in the controls) or to the emergency department (5.7% and 7.3% respectively) because of a fall.

The result regarding fractures was unexpected, with a significantly higher risk in the intervention group than among the controls (OR = 2.39, *p* = 0.023).

Interventions for the prevention of falls in older people living in the community are the object of great scientific interest and intense clinical research, including a series of Cochrane reviews aimed at better understanding “what might work and for whom… (while) the evidence story continues to evolve, exploring ever more precise components of these interventions” [28].

A Cochrane review analyzed the effect of multicomponent interventions on fall-related outcomes [24]. Meta-analysis of the four trials with participants selected by high risk of falling at baseline showed a protective effect of these interventions, with the rate of falls decreased by 21% (95% CI 7% to 32%), a result comparable to that of the PREMIO study. The number of people who sustained at least one fall was reduced by 17% (95% CI 10% to 26%).

Another more recent Cochrane review evaluated interventions with an environmental approach to reducing the risk of falling [29]. In their review, the category of interventions to reduce home fall hazards included studies with an assessment, advice and guidance approach similar to one of the components of the PREMIO multicomponent intervention: a healthcare professional visited participants in their own home one or two times, with telephone follow-up; fall hazards were assessed and a problem-solving strategy was proposed. This strategy raised awareness of falling risk, and assistive technologies were provided in some cases. The results of the meta-analysis (9 studies on older people selected for high risk at baseline) were similar to those of the PREMIO study, with the rate of falls reduced by 38% (95% CI 30% to 44%). The proportion of fallers who had at least one fall was reduced by 26% in the meta-analysis (95% CI 15% to 35%).

The third Cochrane review found that the difference related to the effect of exercise (all types) on the rate of falls in trials in which all participants were at an increased risk of falling was 20% (95% CI 12% to 28%). The difference in the number of people experiencing at least one fall (35 studies) was 13% (95% CI 9% to 17%) [30].

Overall, these reviews showed comparable reductions in fall rates but a greater reduction in the number of fallers compared to the PREMIO study. This relatively small reduction in the number of fallers may be tentatively explained by the fact that a large proportion of patients in PREMIO were overweight or obese (three out of four subjects overweight; three out of ten obese). A systematic literature review of studies of community-dwelling older adults found a U-shaped association between BMI and fall risk, with an increased risk for those with extreme values of BMI relative to those with intermediate values [26].

The multivariate statistical analysis showed that the benefit of the multicomponent intervention was not modified by external factors. For example, the reduction in the mean number of falls in the intervention group is consistent across all age-classes.

Few studies in the Cochrane reviews reported on falls requiring medical attention or hospitalization, yielding uncertain evidence that multicomponent interventions, exercise and home fall-hazard interventions made little or no difference, as in PREMIO [24,29,30,31].

Our unexpected finding of an increased risk of fracture in the multicomponent-intervention group compared with the controls is relevant from a clinical, quality-of-life and socioeconomic perspective. There is a paucity of data and low certainty in the literature on this topic.

Both the Cochrane reviews of home fall-hazard interventions and of the effect of exercise found that such interventions made little or no difference in the risk of people experiencing a fall-related fracture compared with usual care [29,30], while the review of multicomponent interventions included only two trials with one fracture event each, making it inconclusive [24].

In addition, in a component-network meta-analysis of interventions to prevent falls and fall-related fractures in community-dwelling older adults, no single intervention was associated with a reduction in the number of fall-related fractures, while one of the intervention components, assistive technology, was significantly associated with an increase in the number of fall-related fractures (RR 1.66; 95% CI 1.07–2.59) [32].

Finally, in a large trial conducted in the UK among community-dwelling older adults, the fracture-rate ratio was 1.20 (95% CI 0.91 to 1.59) for exercise and 1.30 (95% CI, 0.99 to 1.71) for multifactorial fall prevention compared with mailed advice [33]. No explanation was provided by the authors of that study.

Bone health and fall prevention are often considered separately in public-health settings. The PREMIO study was designed to address the individual risk of falls, not the risk of fractures. A possible explanation for the unexpected increased risk of fractures in PREMIO could be a differential distribution of patients by fracture-related conditions between the intervention and control groups. A higher prevalence of subjects with arthritis/arthrosis was observed in the intervention group (87.2% vs. 83.8%, *p* < 0.05), and this condition is often associated with osteoporosis and higher risk of fractures [34]. Bone mineral density and osteoporosis were also not taken into account, as in other similar studies, although their association with fractures, falls and fear of falling is well known. Another possible explanation is that overtraining or increased confidence may lead to a higher risk of serious consequences of falls. A detailed analysis of all fracture events is planned to investigate this finding in more detail.

The PREMIO study was conducted at the practice level, involving GPs from all over the country, with the aim of incorporating a multifactorial fall-prevention intervention as a regular procedure for each GP. In Italy, general practice is managed by the National Health System, and the relationships between the GP and their patients are therefore quite stable over the patients’ lifetimes, creating an excellent opportunity for this kind of intervention. In PREMIO, the high rate of record completion (72%) demonstrated the possibility of involving primary care professionals in research, linking routine clinical practice to science. The heterogeneity resulting from such a broad and fragmented approach was addressed with methodological tools such as block randomization at the GP level. The high score on the Likert scale used to assess the acceptability of the intervention to patients showed that this type of approach is highly appreciated by older subjects living in the community.

The study has several strengths, starting with the randomized and controlled design, the nationwide distribution of investigators, and especially the practice-oriented intervention model, which was designed to be systematically offered to elderly patients in the practice who were at risk of falling.

Limitations include an overly high expectation of the frequency of falls, which, together with the number of patients who withdrew before the start of the study, reduced the statistical power and resulted in non-significant findings for some of the secondary endpoints. The use of non-probability samples may have increased the risk of sampling bias. Nevertheless, the relatively large number of patients, the wide geographical distribution and the randomized study design provided reliable and relatively robust quantitative estimates that can help guide future studies or public-health interventions in terms of the best strategies to prevent falls among the elderly, especially at home. Furthermore, the lack of a longer follow-up did not allow the authors to evaluate the duration of the benefits and adherence to the intervention program in the long term.

Additional risk factors for falls should be considered in future studies. Such risk factors include substance abuse and excessive alcohol consumption [25]. Smartphone use is also becoming ubiquitous among the elderly. There is a gap in scientific knowledge regarding the risk of fall and neurological and orthopedic disorders associated with smartphone use, but some studies show that it can slow movement, induce systematic imbalance and alter turning behavior, thereby increasing the risk of falling [35,36].

## 5. Conclusions

The PREMIO study, which was based on the classic working model of general practice, demonstrated the ability of GPs to manage intervention studies with large numbers of patients.

The study showed a significant reduction in falls by one in four falls and up to 31% fewer falls at home, although the reduction in the number of fallers was limited. Among the secondary endpoints, admissions to a general hospital or to the emergency room and visits to the GP’s clinic showed marginal improvements. The increased risk of fractures in the intervention group was unexpected and warrants further analyses, given the paucity and uncertainty of published evidence on this topic.

In conclusion, this study confirms that a multicomponent intervention at the primary-care level has the potential to prevent a significant proportion of domestic falls among community-dwelling older people at high risk of falling. The involvement of the GPs is critical because of their role in monitoring adherence to the program. The secondary benefits of the intervention are more difficult to interpret because the study was not specifically designed to measure these outcomes. Non-significant benefits in terms of hospital and emergency-department admissions were offset by an increase in fractures in the intervention group, a finding that may be related to residual confounding after randomization.

Future studies and public-health interventions to prevent falls could benefit from a multicomponent approach including medication review, physical exercise and home risk assessment and should include assessment of risk factors for fractures.

## Figures and Tables

**Table 1:** Distribution of PREMIO study groups by selected individual characteristics.

	Total *n* (%)	Controls *n* (%)	Intervention *n* (%)	*p* Value
	1757	882 (50.2%)	875 (49.8%)	
Sex				
Male	641 (36.5%)	329 (37.3%)	312 (35.7%)	
Female	1116 (63.5%)	553 (62.7%)	563 (64.3%)	*p* > 0.05
Age mean (SD)	77.5 (7.2)	77.4 (7.2)	77.6 (7.3)	*p* > 0.05
Age Class				
65–70 years	348 (19.8%)	180 (20.4%)	168 (19.2%)	
71–80 years	821 (46.7%)	414 (46.9%)	407 (46.5%)	
81–90 years	509 (29.0%)	252 (28.6%)	257 (29.4%)	
90 years	79 (4.5%)	36 (4.1%)	43 (4.9%)	*p* > 0.05
Smoking habit				
Non-Smoker	617 (67.1%)	327 (70.2%)	290 (64.0%)	
Former Smoker	145 (15.8%)	71 (15.2%)	74 (16.3%)	
Current Smoker	157 (17.1%)	68 (14.6%)	89 (19.6%)	
Missing	838 (47.7%)	416 (47.2%)	453 (48.2%)	*p* > 0.05
BMI mean (SD)	28.2 (4.8)	28.2 (4.5)	28.2 (5.1)	*p* > 0.05
BMI Classes				
<18.5 (Underweight)	10 (0.9%)	6 (1.1%)	4 (0.8%)	
18.5–24.9 (Normal weight)	259 (24.5%)	113 (21.4%)	146 (27.6%)	
25–29.9 (Overweight)	477 (45.2%)	253 (48.0%)	224 (42.3%)	
>29.9 (Obese)	310 (29.4%)	155 (29.4%)	155 (29.3%)	*p* > 0.05

**Table 2:** Distribution of PREMIO participants by fall risk factors (inclusion criteria: ≥5 risk factors).

	Total *n* (%)	Controls *n* (%)	Intervention *n* (%)	*p* Value
	1757	882 (50.2%)	875 (49.8%)	
Number of risk factors mean (SD)	6.0 (1.25)	5.9 (1.2)	6.1 (1.2)	*p* > 0.05
Risk Factors				
Use of drugs that increase fall risk	1642 (93.5%)	821 (93.1%)	821 (93.8%)	*p* > 0.05
Cerebro- or cardiovascular diseases	1539 (87.6%)	771 (87.4%)	768 (87.8%)	*p* > 0.05
Polypharmacy	1504 (85.6%)	757 (85.8%)	747 (85.4%)	*p* > 0.05
Arthritis/arthrosis	1502 (85.5%)	739 **(83.8%)**	**763 (87.2%)**	***p <*** **0.05**
Altered vision	1009 (57.4%)	502 (56.9%)	507 (57.9%)	*p* > 0.05
Impaired mobility	799 (45.5%)	387 (43.9%)	412 (47.1%)	*p* > 0.05
Previous falls	798 (45.4%)	382 (43.3%)	416 (47.5%)	*p* > 0.05
Difficulty in knee extension	733 (41.7%)	368 (41.7%)	365 (41.7%)	*p* > 0.05
Creatinine clearance < 65 mL/min	375 (21.3%)	185 (21.0%)	190 (21.7%)	*p* > 0.05
Mental confusion	365 (21.3%)	184 (20.9%)	181 (20.7%)	*p* > 0.05
Social isolation	263 (15.0%)	133 (15.1%)	130 (14.9%)	*p* > 0.05

**Table 3:** Distribution of patients according to selected characteristics and study-related activities of their general practitioners.

	Total *n* (%)	Control Group *n* (%)	Intervention Group *n* (%)	*p* Value
Age mean (SD)	59.4 (4.4)	59.4 (4.4)	59.3 (4.4)	*p* > 0.05
Sex				*p* > 0.05
Male	1342 (76.4%)	670 (76.0%)	672 (76.8%)	
Female	415 (23.6%)	212 (24.0%)	203 (23.2%)	
Patient base				*p* > 0.05
≤1500	807 (45.9%)	407 (46.1%)	400 (45.7%)	
1500	950 (54.1%)	475 (53.9%)	475 (52.3%)	
Records completion	71.6 (11.1)	71.6 (11.1)	71.6 (11.1)	*p* > 0.05
Visits at home				
1			163 (18.6%)	
2			712 (81.4%)	
Telephone calls				
None			174 (19.9%)	
1–11			97 (11.1%)	
12			604 (69.0%)	
Patients enrolled mean (SD)	15.0 (6.1)	7.5 (3.3)	7.5 (3.1)	*p* > 0.05

**Table 4:** Frequency of falls in the last 12 months in the PREMIO study groups in different settings.

	Control	Intervention	Δ Value (Change %)	*p* Value
Mean number of falls				
Outdoor falls mean (SD)	0.47 (1.34)	0.39 (1.19)	−0.08 (−17.0%)	*p* > 0.05
Domestic falls mean (SD)	0.80 (2.01)	0.55 (1.58)	**−0.25 (−31.2%)**	***p <*** **0.02**
All falls mean (SD)	1.27 (2.70)	0.94 (2.20)	**−0.33 (−26.0%)**	***p <*** **0.02**
Patients falling at least once				
Outdoor falls n (%)	92 (14.8%)	84 (13.9%)	−0.9%	*p* > 0.05
Domestic falls n (%)	131 (21.1%)	104 (17.2%)	**−3.9%**	***p =*** **0.05**
All falls n (%)	179 (28.8%)	158 (26.2%)	−2.6%	*p* > 0.05

**Table 5:** Risk of direct consequences of falls by PREMIO study group.

	Patients *n* (%)	Β (SE)	Odds Ratio (95% CI)	*p* Value
Fracture consequent to fall				
Control Intervention	10 (1.6%) 23 (3.8%)	- 0.869 (0.384)	1.00 **2.39 (1.12–5.06)**	***p =*** **0.023**
Hospital admission				
Control Intervention	23 (2.6%) 20 (2.3%)	- −0.147 (0.310)	1.00 0.86 (0.47–1.59)	*p* > 0.05
Emergency room admission				
Control Intervention	64 (7.3%) 50 (5.7%)	- −0.265 (0.195)	1.00 0.77 (0.52–1.13)	*p* > 0.05
Visit to GPs clinic				
Control Intervention	154 (17.5%) 136 (15.5%)	- 0.014 (0.182)	1.00 1.01 (0.71–1.45)	*p* > 0.05

## Data Availability

The data presented in this study are available on request from the corresponding author. The data are not publicly available due to privacy restrictions.

## Supplementary Materials

The following supporting information can be downloaded at: https://www.mdpi.com/article/10.3390/jcm12227134/s1, Supplementary Table S1. Risk of falling associated to the use of selected drugs known to increase the risk of falling. Supplementary Table S2. Description of the physical activity prescribed as part of the intervention. Supplementary Table S3. Evaluation grid of falling risk environmental factors, to be filled by the General Practitioner. Supplementary Table S4. Monthly fall diary to be completed by participants and sent to the General Practitioner. Supplementary Table S5. Monthly diary of physical activity to be completed by participants and sent to the General Practitioner. Supplementary Table S6. Dietary Recommendations. Supplementary Table S7. Customer satisfaction questionnaire completed by all participants. Figure S1. Patients flow.
