# Supplementary material for: A Multicomponent Primary-Care Intervention for Preventing Falls in Older Adults Living in the Community: The PREMIO Study
Source: J Clin Med. 2023 Nov 16;12(22):7134. doi: 10.3390/jcm12227134 (PMC10672084; doi:10.3390/jcm12227134)
Supplement: Supplementary file 1 [file jcm-12-07134-s001.zip › jcm-2621282-supplementary.pdf]

Supplementary Table S1. Risk of falling associated to the use of selected drugs known to increase the risk of falling.

Drugs associated to the risk of falling	Odds Ratio 95% CI
Laxatives	2,03 (1,52-2,72)
Psychotropic drugs	1,74 (1,56-1,95)
Benzodiazepines	1,61 (1,35-1,93)
Antidepressants	1,59 (1,43-1,75)
Antiepileptics	1,56 (1,28-1,90)
Antiparkinson drugs	1,55 (1,21-1,97)
Hypnotics	1,53 (1,40-1,68)
Digoxin (Digitalis glycosides)	1,48 (1,11-1,99)
Narcotics	1,43 (1,27-1,61)
Tranquilizers	1,42 (1,21-1,67)
Metabolic and endocrine drugs	1,39 (1,20-1,62)
Antipsychotics	1,37 (1,16-1,61)
Analgesics	1,33 (1,07-1,65)
Anti-inflammatory drugs	1,25 (1,11-1,42)
ACE inhibitors	1,21 (1,15-1,28)
Vasodilators	1,12 (1,04-1,21)
Antihypertensives	1,10 (1,05-1,16)

Supplementary Table S2. Description of the physical activity prescribed to the intervention group (in Italian in the original document).

RECOMMENDED EXERCISES (BOOKLET "THE 5 EXERCISES OF HEALTH - SIMPeSV")

It is crucial to keep in mind the importance of a very gradual increase in training time and intensity.

Starting position: seated, straight back off the backrest, feet planted on the floor, arms forward;
Movement 1: Lift upper limb with elbows well extended, first the right and then the left. Minimum 5 times;
Movement 2: Bring arms forward and bring arms out. Minimum 5 times;
Movement 3: Bring hands to shoulders with elbows out, raise right arm first and then left arm. Minimum 5 times.

Starting position: seated, back firmly against the backrest, hands on the armrests;
Movement 1: Extend knee, first right then left. Minimum of 5 times;
Movement 2: Extend both knees with toes pointing up and then bring them back to the floor. Minimum 5 times;
Movement 3: Extend both knees with toes pointing up, bring knees toward torso. Minimum 5 times.

Starting position: standing with back straight and hands resting on the back of the chair;
Movement 1: Stand up on tiptoes and return to starting position. Minimum 10 times;
Movement 2: Raise right knee, then raise left knee. Minimum 5 times;
Movement 3: Bring right leg to the side, then bring left leg to the side. Minimum 5 times.

Starting position: sitting, feet planted on the floor, hands firmly on the arms of the chair;
Movement: bring torso forward, push with arms and legs, rise slowly and gradually then..move forward 2-3 steps, then back 2-3 steps. Stand with the back of the legs in front of the chair, grasp the armrests, bring torso forward, accompany the descent slowly and gradually. Repeat at least 3 times.

Starting position: seated, back off the backrest, feet planted on the floor slightly apart, arms off the armrests;
Movement 1: Bring arms forward well outstretched and join hands, then flex torso forward trying to touch the floor. Minimum 3 times.
Movement 2: Flex the torso first to the right, then to the left trying to touch the feet. Minimum 3 times.

OPTIONAL EXERCISES:

Below you can find a table with recommended walking times that can be increased depending on your functional characteristics.

The schedule includes 5 minutes of slow walking at the beginning and end and increasing times of fast walking.

Weeks	Slow walk	Fast walk	Slow walk
1-3	5’	5’-10’	5’
4-6	5’	10’-20’	5’
7-9	5’	20’-30’	5’
From 10° on	5’	30’	5’

Additional recommendations to the patient:

- Store frequently used items in places that are easy to reach without resorting to the use of stools or ladders;
- Always keep drawers closed so as not to trip over them;
- Store clothes in drawers no lower than your knees or higher than your chest;
- Do not wear too long or loose clothes.

Supplementary Table S3. Monthly falls diary to be filled in by participants and to be sent to the General Practitioner (in Italian in the original document)

TO BE COMPLETED BY THE PATIENT AND GIVEN TO THE PHYSICIAN

MONTH ……………………………………………

DATE	FALLS AT HOME		OUTDOOR FALLS		DID THE FALL RESULT IN A VISIT TO YOUR DOCTOR OR SPECIALIST?		DID THE FALL RESULT IN FRACTURE?		DID THE FALL RESULT IN HOSPITALIZATION?	
	YES	NO	YES	NO	YES	NO	YES	NO	YES	NO
	YES	NO	YES	NO	YES	NO	YES	NO	YES	NO
	YES	NO	YES	NO	YES	NO	YES	NO	YES	NO
	YES	NO	YES	NO	YES	NO	YES	NO	YES	NO
	YES	NO	YES	NO	YES	NO	YES	NO	YES	NO

Supplementary Table S4. Monthly diary of physical activity to be filled in by participants and to be sent to the General Practitioner (in Italian in the original document)

MONTH ………………………………………..

**DATE**	**COMPLIANCE** TO PHYSICAL PRACTICE		TOTAL TIME DEVOTED TO PHYSICAL PRACTICE	**DATE**	**COMPLIANCE** TO PHYSICAL PRACTICE		TOTAL TIME DEVOTED TO PHYSICAL PRACTICE
01/01/2016	YES	NO		16/01/2016	YES	NO	
02/01/2016	YES	NO		17/01/2016	YES	NO	
03/01/2016	YES	NO		18/01/2016	YES	NO	
04/01/2016	YES	NO		19/01/2016	YES	NO	
05/01/2016	YES	NO		20/01/2016	YES	NO	
06/01/2016	YES	NO		21/01/2016	YES	NO	
07/01/2016	YES	NO		22/01/2016	YES	NO	
08/01/2016	YES	NO		23/01/2016	YES	NO	
09/01/2016	YES	NO		24/01/2016	YES	NO	
10/01/2016	YES	NO		25/01/2016	YES	NO	
11/01/2016	YES	NO		26/01/2016	YES	NO	
12/01/2016	YES	NO		27/01/2016	YES	NO	
13/01/2016	YES	NO		28/01/2016	YES	NO	
14/01/2016	YES	NO		29/01/2016	YES	NO	
15/01/2016	YES	NO		30/01/2016	YES	NO	
				31/01/2016	YES	NO	

**TOTAL DAYS OF PHYSICAL ACTIVITY**	

**TOTAL MINUTES OF PHYSICAL ACTIVITY**	

Supplementary Table S5. Dietary recommendations (in Italian in the original document)

1. Importance of good hydration	The advice is to drink at least one and a half liters of water daily1, especially during hot times of the year or if we live in very heated environments.<br>For the elderly, it is especially important to drink, regardless of the thirst stimulus, which is felt less.
2. Moderate consumption of wine/alcohol/sweetened beverages	Wine consumption should not exceed one glass per day for women and two glasses per day for men; this recommendation takes into account a possible protective effect against coronary artery disease2.<br>Beer or other alcoholic beverages can be taken as an alternative to wine, but in the case of spirits (liqueurs, schnapps, or bitters) it is necessary to decrease the amount.<br>Sugary drinks are high-calorie foods, not surprisingly they are also often referred to as energy drinks.<br>Fruit juices should also be avoided, because although they contain a percentage of fruit they are often sweetened.<br>Although sugary drinks provide energy, they do not satiate nor reduce the intake of other caloric foods, so as a result they result in an excessive intake of calories that will lead those who consume them to weight gain2.<br>Obesity is considered a disease and is a risk factor for other diseases including diabetes, dyslipidemia, and cardiovascular disease.
3. Avoid complete meals with first, second and side dish	It is better to opt for a balanced single dish that contains all the nutrients of an entire meal.<br>Some of the possible combinations are pasta with legumes and/or vegetables, meat/fish/eggs with vegetables.<br>The tendency to eat too many dishes in a single meal occurs especially when traveling, staying in a hotel or restaurant1.
4. Season with olive oil and iodized salt	Raw or cooked vegetables, preferring to be cooked in a way that maintains mineral and vitamin intake, can be seasoned with raw olive oil1.<br>Salt addition should be moderate and it is preferable to use iodized salt, especially for those living in regions far from the sea.
5. Eat while sitting at the table making sure to chew carefully	For proper assimilation of food, it is best to eat the main meals of the day while sitting at the table, making sure to chew carefully; part of the digestive process already takes place in the mouth through the contact of food with saliva.<br>There should be at least three meals in the day, and the most important one is breakfast because it comes after the longest fasting period in the 24-hour period, and provides the "fuel" for the whole day.<br>Not eating an adequate breakfast also predisposes to higher caloric intake during the rest of the day1.

6. Importance of a varied diet and caution not to vary too much in weight	Obesity can be considered in effect a disease, and to prevent it, the ideal is to follow a varied and equillibrate diet3.<br>However, excessive weight loss should also cause concern.<br>Elderly individuals in general, and in particular those with malignant disease, chronic obstructive pulmonary disease (COPD), gastrointestinal disease, those on polypharmacy, and those living alone, may be at risk of malnutrition with or without sarcopenia, with loss of lean mass (muscles, skeleton and organs such as the heart, brain and liver, blood cells and immune system).
7. Increase consumption of seasonal fruits and vegetables	Each day an adult should consume 5 servings (at least 400-600 grams) of non-starchy vegetables2 (excluding potatoes) and fruits, varying the type by also choosing according to the colors of the vegetables (red, green, yellow, white, violet and orange..).<br>Dried fruits (nuts, hazelnuts, almonds), although caloric and therefore to be eaten in moderation, are an important source of nutrition2.<br>Foods of plant origin, and not those of animal origin, should constitute the food around which the meal is built.
8. . Balance meat/fish consumption	Meat and fish are foods that provide protein, however, when choosing meat, leaner cuts are preferred to reduce fat intake3.<br>It is advisable to consume 4-5 servings of meat per week (one serving is considered equal to 100 g of meat).<br>Fish and seafood are rich in omega-3, nutrients that introduced into the diet are protective factors for cardiovascular disease.<br>It is recommended that fish be eaten at least twice a week, boiled or roasted, thus using forms of cooking that do not add fat4.<br>Foods such as sausages, fatty meats and fish, and offal should be limited because of their higher fat content4.
9. Balance egg and legume consumption	Eggs are protein-rich foods, but their consumption over the week should be limited because of their high cholesterol content. It is appropriate to eat no more than two eggs per week and cook them without added fat (soft-boiled, hard-boiled).<br>Legumes are among vegetables those with the highest protein content; they are also important as sources of B vitamins (B1 and B2), niacin and folate.<br>They also provide mineral salts, containing fair amounts of iron, zinc and calcium minerals (zinc, copper, highly bioavailable iron, etc.), some B3 complex vitamins.<br>They are also foods rich in fiber, nutrients that help intestinal transit.<br>It is recommended to eat at least twice a week a portion of legumes (equal to about 30 g of dried legumes or 100 g of fresh legumes)4
10. Balancing milk and dairy products	Sometimes milk and dairy products tend to be eliminated from the diet because they are considered fatty foods that lead to weight gain, however, there are many low-fat dairy products with no added sugar3 (skim or semi-skimmed milk and yogurt, fresh and low-fat cheeses) that you should keep in your diet.<br>It is advisable to avoid the use of cream and butter4 in cooking.<br>In fact, milk and dairy products are important for calcium intake and if not taken should be adequately replaced by other calcium-containing foods to avoid especially diseases related to bone demineralization4.

- *Check food labels to evaluate expiration and storage methods*

Salt consumption in the diet should be reduced.

Foods that are in the form of canned foods often contain a lot of salt, and therefore should be avoided.

Preservation methods that do not involve the use of salt include refrigeration, freezing, bottling, sterilization, and fermentation.

Reducing salt helps keep blood pressure low and thus decreases the risk of cardiovascular disease 5.

In addition, by progressively salting foods less, and using spices to flavor foods, our palate gets used to and will continue to feel and appreciate flavors distinctly.

It is important not to consume foods or foods after the expiration date on the package, and especially be careful not to consume dried grains or legumes that are moldy or poorly stored1, 5.

- *How to keep good resolutions?*

The first thing to do is to become well informed about why it is important to change an aspect of your diet or lifestyle; in fact, an informed person is likely to be more motivated to change.

It is important, if you do not live alone, to involve your family members, especially in the area of nutrition, for it is difficult to eat healthy if the rest of the family binges on sweets and fried foods.

"Slow and steady wins the race," says the proverb; introducing change gradually is the best way to maintain new habits over time.

With your doctor, you can establish a program with increasing commitment, and you can evaluate your progress together 1.

1- *Decalogo Alimentazione estate 2012 E…state OK con la nutrizione, Ministero della Salute*

2- *Food, Nutrition, Physical Activity, and the Prevention of Cancer, a Global Perspective, Washington D.C. 2007, Rapporto del World Cancer Research Fund International*

3-*Dietary Guidelines for Americans 2005 U.S. Department of Health and Human Services U.S. Department of Agriculture www.healthierus.gov/dietaryguidelines*

4- *Piramide alimentare Italiana, una guida settimanale per uno stile di vita salutare a cura dellea Sezione di Scienza dell'Alimentazione Dipartimento di Fisiopatologia Medica SAPIENZA Università di Roma - www.piramidealimentare.it*

5- *Diet, nutrition and the prevention of chronic diseases - report of the joint WHO/FAO expert consultation 2004*

Supplementary Table S6. Evaluation grid of fall risk environmental factors. To be filled by the General Praticitioner (in Italian in the original document)

**HAZARD**	**Tick if present**	**SUGGESTIONS**
Slippery flooring		Do not wax
Carpet laid on slippery floor		Use non-slip nets or remove carpet if not necessary
Long-pile carpet		Remove
Uneven, chipped or peeling flooring		Repair
Insulated steps or raised thresholds		Mark obstruction
Wall cabinets and bookcases not securely fastened to wall		Fix them
Furniture with protruding base or feet		Mark obstruction
Doormat of excessive thickness		Replace
Door does not open fully		Move movable obstacles
Furniture that excessively reduces the width of the pathway		Move the furniture so that the min passage is 60 cm
Dangerous coincidence of simultaneous opening of several doors		Change direction of opening or affix markings
Poor or misplaced lighting		Enhance lighting<br>or arrange new lighting points
Absence of handrails along stairs		Install
Ramp of stairs in front of entrance		Modify or mark
Light control far from bed		Install in easily accessible location
Absence of non-slip mats in the shower stall or bathtub		Equip with suitable mats
Mats near bathroom fixtures		Eliminate or remove them immediately after use
Absence of grab bars in the tub or in the shower		Install
Presence of folding chairs		Eliminate

Supplementary Table S7. Customer satisfaction questionnaire filled in by all participants (in Italian in the original document)

Instructions - Reflect on your level of satisfaction or dissatisfaction with the project that was proposed to you in this trial. We would like your opinion on the effectiveness, practicality of the educational intervention. For each question, mark only the answer that best corresponds to your experiences.

1. How satisfied or dissatisfied are you with the project's ability to prevent or treat the risk of falls at home?

1 Extremely dissatisfied
2 Very dissatisfied
3 Dissatisfied
4 Moderately satisfied
5 Satisfied
6 Very satisfied
7 Extremely satisfied

2. How convenient or inconvenient was it to follow the instructions for environmental remediation?

1 Extremely uncomfortable
2 Very uncomfortable
3 Uncomfortable
4 Relatively comfortable
5 Comfortable
6 Very comfortable
7 Extremely comfortable

3. How comfortable or uncomfortable was it to follow the instructions for the physical activity program?

1 Extremely uncomfortable
2 Very uncomfortable
3 Uncomfortable
4 Relatively comfortable
5 Comfortable
6 Very comfortable
7 Extremely comfortable

4. Overall, how confident are you that the project benefited you?

1 Not at all confident
2 Slightly confident
3 Fairly confident
4 Very confident
5 Extremely confident

5. How confident are you that the advantages offered by the project outweighed the disadvantages?

1 Not at all sure
2 Not very sure
3 Fairly sure
4 Very sure
5 Extremely secure

6. All in all, how satisfied or dissatisfied are you with the project in general?

1 Extremely dissatisfied
2 Very dissatisfied
3 Dissatisfied
4 Moderately satisfied
5 Satisfied
6 Very satisfied
7 Extremely satisfied

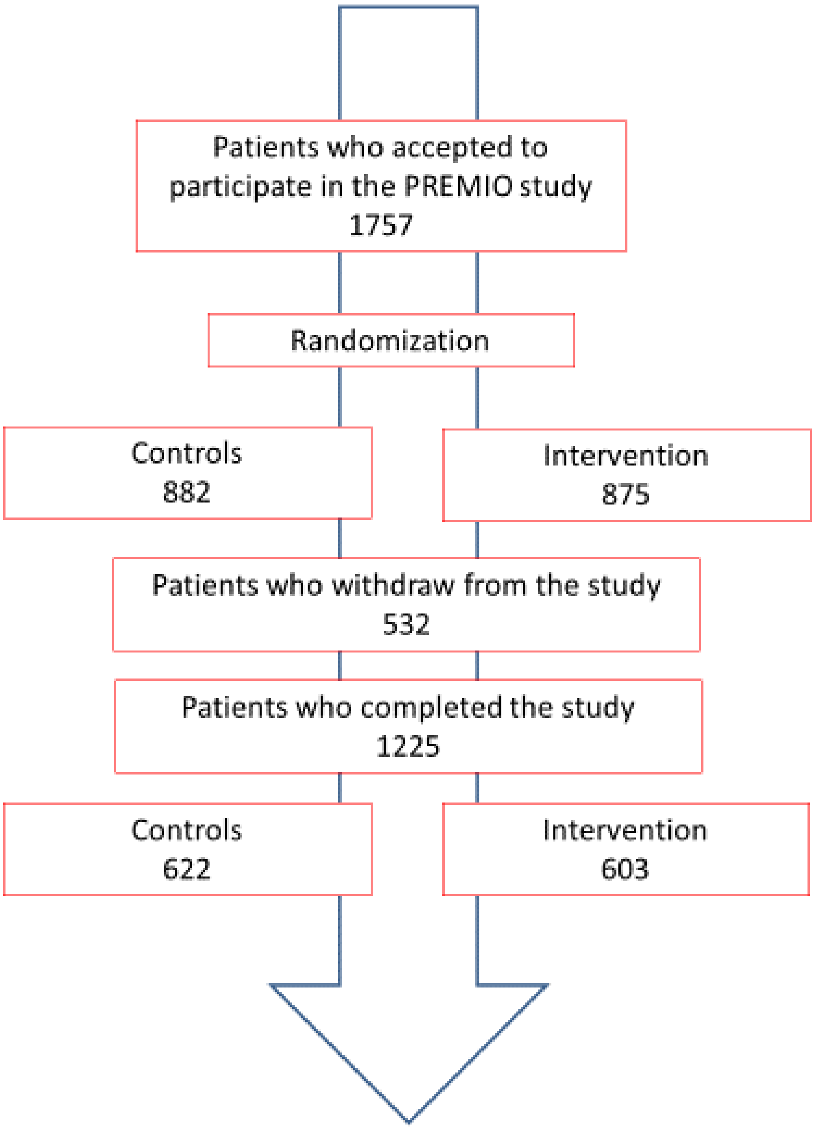

Figure S1. Patients flow.
